# Myo-inositol increases respiratory burst, cellular proliferation, and phagocytosis of cultured leukocytes from Nile tilapia

**DOI:** 10.1016/j.cirep.2024.200193

**Published:** 2024-12-27

**Authors:** Edgar Junio Damasceno Rodrigues, Pedro Luiz Pucci Figueiredo de Carvalho, Vitor Fernandes Silva, Delbert M. Gatlin, Margarida Maria Barros

**Affiliations:** a Faculdade de Medicina Veterinária e Zootecnia, Departamento de Melhoramento e Nutrição Animal, Universidade Estadual Paulista “Júlio de Mesquita Filho”, São Paulo, Brazil; b Department of Ecology and Conservation Biology Texas A&M University, College of Agriculture and Life Sciences, College Station, TX, USA; c AQUOS ‐ Aquatic Organisms Health Laboratory, Department of Aquaculture, Federal University of Santa Catarina (UFSC), Florianópolis, Santa Catarina, Brazil

**Keywords:** Immunomodulation, Additive, Aquaculture

## Abstract

Myo-inositol (MI) is a functional nutrient known to positively affect growth and health parameters when supplemented in appropriate concentrations. This bioactive molecule plays an essential role by modulating several signaling pathways including those related to some immunological responses. Therefore, this study aimed to evaluate the effects of MI supplementation on immunological responses of Nile tilapia head-kidney-derived leukocytes. Primary cell cultures of head-kidney leukocytes were used to evaluate respiratory burst, cell proliferation, and phagocytosis. Five concentrations of MI were supplemented to the culture media: 0, 300, 600, 900, and 1200 mg l^-1^ with 16 replicates per concentration. MI supplementation at 900 mg l^-1^ increased intracellular anion superoxide production up to 15 %, whilst 1200 mg l^-1^ boosted extracellular anion superoxide production up to 39 % in comparison with the basal group. The phagocytic capacity of cultured cells supplemented with 1200 mg l^-1^ MI was improved by 24 % in comparison with the basal group. Proliferative responses also were enhanced by approximately 47 % when cells were supplemented with 900 mg l^-1^ of MI. Quadratic regression analysis determined 872 mg l^-1^ to be the optimum supplementation level of MI for leukocyte proliferation. Based on the results of all measured responses, 900 mg l^-1^ of MI was considered the optimal supplementation level for stimulating immunological functions of cultured Nile tilapia leukocytes. The findings of this study provided evidence of both the positive effects of MI and its role in modulating immunological responses of leukocytes highlighting MI potential as an immunomodulatory aquafeed additive.

## Highlights

• Myo-inositol (MI) increased nile tilapia cultured leukocytes activity.
• Intracellular anion superoxide production by isolated leukocytes was boosted by MI.
• Extracellular anion superoxide produced by isolated leukocytes was increased by MI.
• Isolated phagocyte activity was improved by MI supplementation.
• Cultured leukocyte proliferation was enhanced by MI culture-media enrichment.

## Introduction

The intensification of aquaculture production in various types of intensive systems challenges fish welfare and health. To prevent and/or treat disease outbreaks in aquaculture, antimicrobials have been frequently, and in some cases, inappropriately used. The utilization of such antimicrobials as a strategy to mitigate the occurrence of infectious diseases is still a noted practice in aquaculture and represents a risk of selecting resistant strains [1]

As an alternative, functional feed additives with immunomodulatory properties have been considered potential substitutes for antimicrobials in disease prevention, in addition to potentially increasing the immune resistance of aquatic animals and, consequently, supporting the sustainable development of the aquaculture industry. Pre and probiotics, glucans, animal and plant extracts, enzymes, and enzymatic blends are examples of feed additives with immunomodulatory properties used in fish nutrition. In fact, different authors verified the positive effects of those additives on immunological parameters, [2,3] antioxidant system [4], disease resistance and growth performance [5,6].

In this regard, myo-inositol (MI) is considered an important nutrient, with different functions in cellular metabolism and a high potential for use as a feed additive in animal nutrition. Myo-inositol is a constituent of cell membranes, acting in cellular signaling and regulating enzyme activity and lipid transport [7]. Moreover, this carboxylic sugar has been shown to positively modulate different immunological responses in fish, such as inflammatory cytokine regulation [8], reduction of cell apoptosis [9], and increases in lysozyme and alkaline phosphatase mucus concentrations when supplemented in appropriate levels [6]. Such effects are associated with MI participation in cell signaling pathways [10], phosphoinositides production [11], and cyclins A, E, and D activity which are directly regulates cell proliferation [9]. Considering the mechanisms that MI is involved in, supplementing this additive in commercial aquafeeds could increase fish resistance against disease outbreaks and contribute to antimicrobial usage.

In fact, it was observed that in common carp (*Cyprinus carpio* var. Jian) immune responses were positively affected by supplementing MI at 232.7 to 687.3 mg kg^-1^ diet, leading to an increase in phagocytosis index, as well as serum lysozyme activity (Jiang et al. [12]). Inappropriate levels of MI in common carp diets were associated with a reduction in lysozyme and acid phosphatase activities, as well as complement 3 and 4 contents in the head kidney and spleen when results were compared with those obtained under optimal dietary MI levels 770.5 mg kg^-1^ [9]. The deficiency of this nutrient was further responsible for poor productive performance in hybrid tilapia (*Oreochromis niloticus* × *O. aureus*) [7], and other fish species as compiled by Cui et al. [10]. Therefore, MI supplementation in commercial diets is a common and necessary practice for some fish species, such as Nile tilapia, considering that its endogenous production is not enough to meet the metabolic requirements for optimal growth ( [[7], [13], [14]]). Notwithstanding, research investigating the effects of dietary MI supplementation on Nile tilapia health is scarce, but promising [15].

Considering the specific characteristics of MI and its important role on the fish immune system, *in vitro* experiments might be an essential tool to understand the complexity of its mechanisms. *In vitro* techniques tend to minimize the external influences promoted by *in vivo* trials and will allow for the assessment of MI effects on specific, isolated components of the fish's immune system. Based on that reasoning, this study aimed to evaluate the effects of MI supplementation in the culture medium of head-kidney-derived leukocytes of Nile tilapia on respiratory burst, phagocytosis and proliferation responses.

## Material and methods

### Fish and culture media

A group of Nile tilapia (100 ± 11.43 g) was housed in a recirculation water system composed of 30, 38-L tanks provided with supplementary aeration, and mechanical and biological filtration. Fish were fed a commercial diet (Rangen®, Angleton, Texas) containing 32 % crude protein and 5 % crude fat twice a day to apparent satiation for 30 days. Before sampling, healthy fish were anesthetized using a tricaine methane sulfonate solution (MS-222, 100 mg L^−1^, Tricaine-S, Western Chemical Inc., Ferndale, WA, USA). Then, blood samples from seven fish were collected with heparinized syringes and used for evaluating neutrophil oxidative radical production based on the reduction of nitroblue tetrazolium (NBT, cat 0329–1 G, VWR) by superoxide anion (O^2-^).

After blood sampling, fish were euthanized (MS-222, 500 mg L^−1^) and head kidneys were aseptically excised and pooled into a homogeneous sample. Then, the head- kidney-derived leukocytes were isolated and cultured according to Secombes [16], with slight modifications. The culture media was prepared according to Carvalho et al. [17]. Complete culture media (CCM) consisted of MI-free Leibowitz cell culture (L-15, REF10–045-CV, Corning) media, 50 units mL^−1^ of penicillin and 0.05 mg mL^−1^ of streptomycin (cat #P0781, Sigma Aldrich) plus 5 % of bovine calf serum (BCS, cat # 12133C, Sigma Aldrich) and served as the control medium. Based on literature considering *in vivo* trials [[7], [12], [14], [15]], CCM was supplemented with MI at 0, 300, 600, 900, or 1200 mg l^-^, resulting in five different media treatments.

### Leukocytes culture

Leukocytes were isolated from the head kidney following the methodology described by Secombes et al. [16], with modifications. Head kidney tissue was sampled and stored in l-15 supplemented with 2 % BCS. After that, samples were homogenized using a glass Potter-Elvehjem tissue grinder and filtered through a 100 μm sterile nylon mesh. The filtered cell homogenate was centrifuged and washed with cold, sterile phosphate buffer saline (PBS, cat #P4417, Sigma Aldrich). Following that, isolated cells were layered on a Percoll (cat #P1644, Sigma Aldrich) gradient (51 % v/v) and centrifuged at 400 × g for 30 min. The cell layer formed was collected and washed twice with ice-cold PBS at 200 × g for 10 min.

The isolated leukocytes were counted using a hemocytometer, and viability was assessed by Trypan blue staining. Cells used in this trial presented a survivability greater than 95 %. The cell homogenate was adjusted to 2 × 10^7^ cells mL^−1^ in l-15 with 0.1 % BCS. After that, 100 μl aliquots of leukocyte suspension were pipetted in each well of sterile flat bottom 96-well microplates, which were incubated at 27 °C for 2 h for cell attachment. Then, the culture media was substituted with MI-supplemented media, and the microplates were incubated overnight for further analysis.

### Respiratory burst

The respiratory burst activity of head-kidney-derived leukocytes was evaluated by measuring intra- and extra-cellular superoxide anion production, as well as whole-blood NBT reduction as described by Secombes [16] and Anderson and Siwicki [18], respectively.

In brief, for the extracellular assay, 100 μl of cytochrome c solution (cat #C2506, Sigma Aldrich) (1.5 mg mL^−1^, in phenol red-free PBS) mixed with phorbol 12-myristate 13-acetate (PMA, cat #P8139, Sigma, 1 μg mL^−1^) was added to 16 wells per treatment. Another set of 16 wells per treatment was used as control, adding 100 μl of cytochrome c solution homogenized with PMA and superoxide dismutase (SOD, 300 U ml^−1^) (cat #S2515, Sigma Aldrich). After that, a plate reader spectrophotometer was used to measure the absorbance at 545 nm every 15 min. Extracellular O^2−^ production was calculated according to Carvalho et al. [17]: nmol of anion superoxide O^2−^ = [(Δ Absorbance after 45 min × 100) ÷ 6.3].

Intracellular production of O^2−^was evaluated by quantifying formazan granules formation. Briefly, 100 μl of nitro blue tetrazolium (NBT, cat #N6876, Sigma Aldrich) 0.1 % diluted in PBS (PBS, cat #P4417, Sigma Aldrich) was added to 16 wells per treatment and incubated at room temperature for 45 min. The isolated leukocytes were washed twice with 100 % methanol (MeOH) and then fixed with 70 % methanol. After that, 120 μl of 2 M KOH and 140 μl dimethyl sulfoxide (DMSO, cat #D8418, Sigma Aldrich) were pipetted into the wells to dissolve formazan crystals. The turquoise-blue solution generated was re-suspended, and absorbance was determined in a plate reader spectrophotometer at 620 nm.

Nitroblue tetrazolium reduction by superoxide anion (O^2-^) generated from phagocytes metabolism in fish blood was measured following the methodology described by Anderson and Siwicki [18] with slight modifications. A 50 μl blood aliquot of each sample was mixed and incubated with 50 μl of CMC for one hour at room temperature. After that, each sample was mixed with 100 μl of NBT solution (0.2 %) and incubated at room temperature for 30 min. Then, 50 μL of that mixture was added to 1 mL of N, N-dimethylformamide (DMF, DX1730–6, Sigma Aldrich) and centrifuged for 5 min at 3000 rpm. Supernatant was collected and optical density was read at 540 nm. The absorbances were converted to mg ml^-1^ as described by [19].

### Phagocytes activity

Cultured Nile tilapia phagocyte activity was assessed by measuring the phagocytosis of stained yeast cells (*Saccharomyces cerevisiae*) as described by Ainsworth and Chen (1990), with modifications. Briefly, 100 µl of leukocyte suspension (1 × 10^7^ cells ml^-1^) was seeded on a sterile flat bottom 96-well microplate and incubated at 27 °C for 2 h to allow cell attachment. The culture media was then removed and substituted with MI-supplemented culture media. After a 24-h incubation period, 10 µl of autoclaved dyed yeast suspension (providing a yeast cell:leukocyte ratio of 10:1) was mixed to the leukocyte suspension and incubated at room temperature for 60 min to allow for phagocytosis. Then, the attached cells were carefully washed three times with room temperature PBS after which 100 µl of trypsin-EDTA solution (1.5 g l^-1^ trypsin and 0.4 g l-1 EDTA in PBS) was added to each well and incubated overnight at 37 °C to solubilize the leukocytes and engulfed yeast, thus resuspending the dye. Absorbance values were read in a plate reader spectrophotometer operating at 620 nm.

### Leukocytes proliferation

The proliferation of head-kidney-derived leukocytes upon stimulation with a non-specific mitogen was measured using the tetrazolium salt 3-(4,5-dimethyl-2 thiazolyl)−2,5-diphenyltetrazolium bromide (MTT) cat #M2128, Sigma Aldrich, 5 mg mL^−1^) followed by the colorimetric assay as described by Mosmann [20], with modifications. Supplemented media were added in sets of 16 wells (for each treatment) of leukocyte primary culture plates. Lipopolysaccharide solution (LPS, cat #L2630, Sigma Aldrich, 0.1 mg mL^−1^) was used to stimulate proliferation. A set of 16 wells did not receive mitogen stimulation and served as a negative control. The resulting plates were incubated for 18 h at 27 °C. Following that, 10 μl of 5 mg MTT mL^−1^ solution was added to each well and incubated at 27 °C for 4 h. Dimethyl sulfoxide was used to dissolve the precipitated formazan crystals, and the optical density was measured at 570 nm using a plate reader. Leukocyte proliferation capacity was computed as stimulation index (SI = ABS stimulated cells ÷ ABS non-stimulated [control] cells).

### Statistical analysis

Data obtained from the *in vitro* assays were tested for homogeneity and normality using Browne-Forsythe and Shapiro-Wilk tests. Subsequently, data were subjected to analysis of variance (ANOVA) with the assistance of Minitab 19 software to determine whether MI supplementation levels significantly (P < 0.05) affected the parameters evaluated. After that, Tukey's test was applied to compare the means (*p* < 0.05).

Additionally, a follow-up trend analysis using orthogonal polynomial contrasts was performed to determine if the significant effects followed linear and/or quadratic patterns. After orthogonal contrast evaluation, proliferative response data was submitted to a second-order polynomial model (*y* = ax^2^ + bx + *c*) to determine the optimal supplementation level of MI.

## Results

### Respiratory burst

**Fig. 1:**
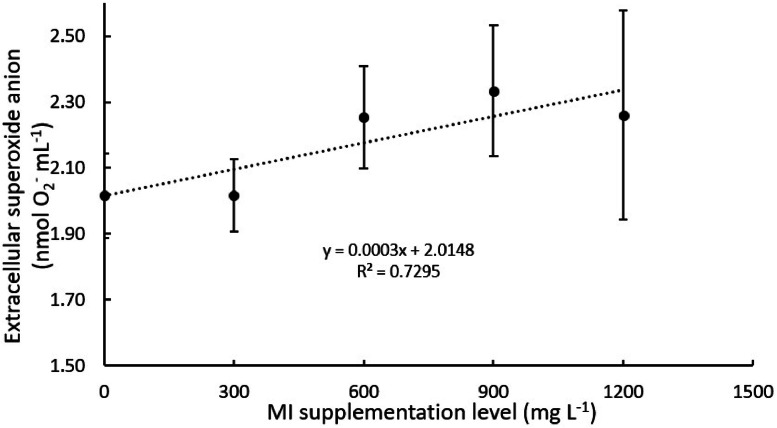
Extracellular superoxide anion production by Nile tilapia head-kidney derived leukocytes incubated with graded levels of myo-inositol (mg l^-1^) Values represent the means of 16 replicate wells.

**Fig. 2:**
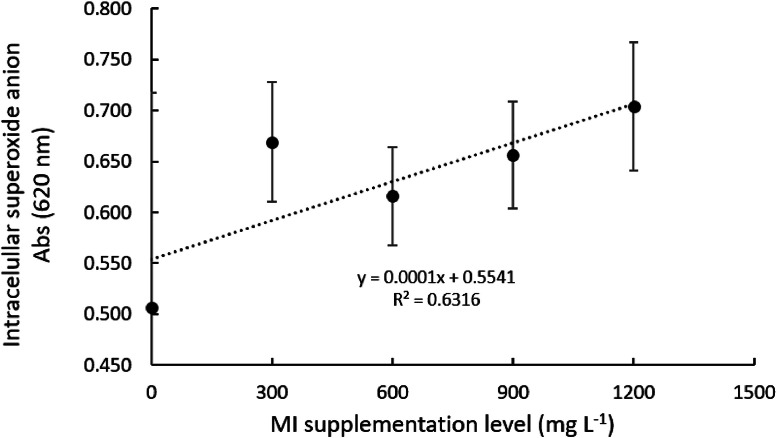
Intracellular superoxide anion production by Nile tilapia head-kidney derived leukocytes incubated with graded levels of myo-inositol (mg l^-1^) Values represent the means of 16 replicate wells.

Overall, the intra- and extra-cellular production of superoxide anion by Nile tilapia head-kidney-derived leukocytes was positively affected by myo-inositol (MI) graded levels in culture media (Table 1; *P* < 0.05). MI supplementation at 900 and 1200 mg l^-1^ significantly increased intra- and extra-cellular anion superoxide production when compared to the non-supplemented group. After subjecting both extra- and intra-cellular anion superoxide production data to a follow-up trend analysis (Fig. 1, Fig. 2, respectively), it was observed that MI supplementation manifested a linear effect (Table 1; *P* < 0.05). Conversely, NBT reduction by Nile tilapia's whole blood leukocytes was not affected (*P* < 0.05) by MI-rich culture media resulting in a reduction of 6,19; 6,33; 6,29; 6.01; 5,91 mg ml^-1^ to 0; 300; 600; 900 and 1200 mg l^-1^ respectively.

**Table 1:** Respiratory burst activity of head-kidney-derived leukocytes incubated with graded levels of myo-inositol (mg l^-1^).

**CCM**	**0**	**300**	**600**	**900**	**1200**	**P-value**	**P.S.E.**	**Linear trend**	**Quadratic** **trend**
**Extracellular (nmol)**	2.01^B^	2.00^B^	2.26^AB^	2.31^A^	2.26^AB^	0.005	0.192	0.0011	0.3090
**Intracellular (Abs)**	507^B^	669^A^	616^A^	656^A^	704^A^	0.000	109	0.0002	0.0946
**Phagocytosis activity (abs)**	0.217 ^B^	0.217 ^B^	0.205^B^	0.253^A^	0.270^A^	0.000	0.031	0.0001	0.0011

Values represent means of 16 replicate wells. Means within the same row that share a common letter do not differ statistically (Tukey's test, *p* < 0.05). CCM, Complete culture media, P.S.E., pooled standard error.

### Leukocytes proliferation capacity

**Fig. 3:**
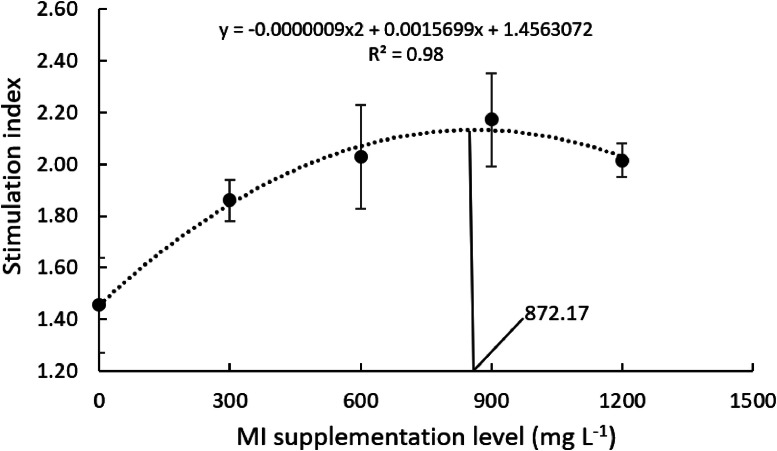
Proliferation of head-kidney-derived leukocytes of Nile tilapia incubated with graded levels of myo-inositol (mg l^-1^) Values represent the means of 16 replicate wells.

The proliferation capacity of Nile tilapia head-kidney-derived leukocytes was positively affected by graded MI levels (Table 2; *P* < 0.05). Supplementing MI at 900 mg l^-1^ to culture media produced a higher proliferation response in comparison with the non-supplemented group. After subjecting cell proliferation capacity to a follow-up trend analysis, a quadratic trend effect of MI supplementation was observed (Table 2; *P* < 0.05). A second-order regression analysis determined 872 mg MI l^-1^ as the optimum supplementation level for optimal cell proliferation (Fig. 3).

**Table 2:** Proliferation capacity upon non-specific stimulation of head-kidney-derived leukocytes incubated with graded levels of myo-inositol (mg l^-1^).

CCM	0	300	600	900	1200	P-value	P.S.E.	Linear trend	Quadratic trend
Head-kidney leukocytes	1.46 ^D^	1.86 ^C^	1.99 ^B^	2.14 ^A^	2.02 ^B^	0.000	0.113	0.0001	0.0000

Values represent means of 16 replicate wells. Means within the same row that share a common letter do not differ statistically (Tukey's test, *p* < 0.05). CCM, Complete culture media, P.S.E., pooled standard error.

### Phagocytosis activity

**Fig. 4:**
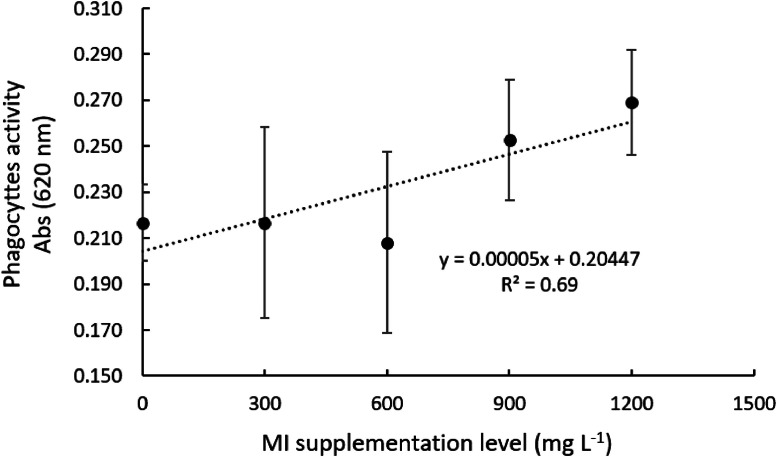
Phagocytes activity of Nile tilapia head-kidney-derived leukocytes incubated with graded levels of myo-inositol (mg l^-1^) Values represent the means of 16 replicate wells.

The phagocytosis activity of Nile tilapia head-kidney-derived phagocytes was positively affected by MI supplementation (Table 2.; *P* < 0.05). Higher levels of MI supplementation (900 and 1200 mg l^-1^) significantly enhanced the phagocytic activity (Table 2. *P* < 0.05) when compared with the other MI levels. A follow-up trend analysis determined a linear effect of MI supplementation on phagocytosis activity (Table 2, *P* < 0.05), as observed in Fig. 4.

## Discussion

Myo-inositol effects on growth performance [[7], [14], [15]], influence on the immunological system ([9], 2010; [[6], [8], [15]]) and physiological functions compiled by Cui et al. [10] of aquatic species have been evaluated. However, the effects of MI on some immunological functions in fish species, including Nile tilapia, require further investigation.

Compared with other vertebrates, fish immunological defense relies mainly on innate responses to eliminate pathogens with subsequent activation of adaptative responses. Respiratory burst represents the activity of leukocytes when eliminating invading microorganisms. After engulfing pathogens, the leukocytes produce several substances such as reactive oxygen species and nitrogen that not only will damage the cellular membrane of pathogenic microorganisms but also trigger adaptative responses depending on the severity of the infection [21]. MI plays a significant role in several immunological mechanisms including phagocytosis, lymphocyte proliferation, and non-specific immunological responses [10]. To our knowledge, there are limited studies evaluating the effects of MI supplementation on respiratory burst, phagocytosis, and leukocyte proliferation of Nile tilapia *in vivo* and *in vitro*. Therefore, this pioneer study evaluated the effects of MI supplementation on the aforementioned parameters.

There are different methodologies to evaluate the respiratory burst of leukocytes. In this study, this parameter was assessed by quantifying extracellular and intracellular superoxide anion production, as well as whole-blood nitro blue tetrazolium (NBT) reduction. Overall, higher levels of MI supplementation to the culture media positively modulated extra- and intra-cellular anion superoxide production of Nile tilapia leukocytes compared with the non-supplemented group. In fact, extra- and intra-cellular anion superoxide production was enhanced by 15 % and 39 % when MI was supplemented at 900 and 1200 mg l^-1^, respectively. Overall, these results corroborate those from the phagocytosis assay in which MI supplementation increased phagocytosis up to 24 % when compared with the non-supplemented culture media. Conversely, the whole blood NBT reduction was not affected by MI supplementation. This result may be related to the methodology used to evaluate the NBT reduction instead of the MI effect on the mentioned parameter. The authors hypothesized that the incubation time of whole blood leukocytes with the chosen MI concentrations might be not enough to promote significant effects on NBT reduction.

Myo-inositol has been associated with enhancements in phagocyte activity of fish (Jiang, et al. [12]) and other animals [22]. It was observed that dietary supplementation of 400 mg kg^-1^ of MI improved Jian carp (*Cyprinus carpio* var. Jian) phagocyte activity against *Aeromonas hydrophila* infection (Jiang, et al. [13]). Accordingly, positive effects of MI supplementation on phagocytosis also were found in the present study and, to the best of our knowledge, are the first reports on the influence of MI on Nile tilapia phagocytic activity. There are different mechanisms in which MI could be involved in the stimulation of phagocytosis. This molecule promotes the depolarization of the leukocyte membrane, increasing the potential membrane difference between the host defense cell and the pathogen, which in turn increases cellular attraction and optimizes phagocytosis [22]. Moreover, myo-inositol is the precursor of biosynthetic components of several phosphoinositides [11]. These phospholipids are involved in plasma membrane distortion promoted by actin polymerization and contraction, thus being essential for pathogen phagocytosis [23].

Besides affecting respiratory burst and phagocytosis, MI supplementation to the culture media of Nile tilapia leukocytes also improved cell proliferation. Such proliferation was most affected by 900 mg MI l^-1^, resulting in an increase of 47 % compared with the non-supplemented cell culture. Similar results were obtained by Lazado et al. [24]. Those authors observed that crude phytase supplementation to head-kidney cultured leukocytes of Atlantic cod (*Gadus morhua*) increased leukocyte proliferation due to phytate hydrolysis by phytase, which yields myo-inositol and other inositol phosphates. Similar associations were described in previous studies with other fish species. The correlation between MI supplementation and leukocyte proliferation was further described for fish species by Jiang et al. [9]. In that study, dietary MI supplementation to juvenile Jian carp up-regulated the gene expression in head-kidney and spleen of the E2F4 signaling factors, cyclins D1, A, and E which are involved in cell proliferation with different degrees and roles. Bearing this in mind, we conclude that this study offers scientific evidence of the importance of MI for Nile tilapia immunological parameters. Further studies including *in vivo* assessments and disease-resistance trials should be performed to consolidate the results found herein and expand our understanding of the immune-boosting characteristics of MI and its potential use in functional and environmentally friendly aquafeeds.

## Conclusions

Overall, myo-inositol (MI) enhanced the immunological activity and proliferation of Nile tilapia cultured leukocytes, and 900 mg l^-1^ is considered the optimum supplementation level to improve phagocytic activity, killing capacity, and cell proliferation of cultured Nile tilapia leukocytes. Considering the findings of this study, MI supplementation positively affected immunological responses evaluated emphasizing that MI could be a promissory aquafeed additive to contribute with increasing fish resistance to disease resistance. Moreover, the results of this *in vitro* study are promising and warrant further studies on MI effects on Nile tilapia's immunological responses using an *in vivo* model.

## Ethics statement

All procedures performed were approved by the Institutional Animal Care and Use Committee at Texas A&M University under IACUC 2022–0162.

## CRediT authorship contribution statement

**Edgar Junio Damasceno Rodrigues:** Writing – review & editing, Writing – original draft, Methodology, Investigation, Formal analysis, Data curation, Conceptualization. **Pedro Luiz Pucci Figueiredo de Carvalho:** Writing – review & editing, Supervision, Methodology, Investigation, Formal analysis, Data curation. **Vitor Fernandes Silva:** Writing – review & editing, Methodology, Investigation, Conceptualization. **Delbert M. Gatlin:** Writing – review & editing, Visualization, Supervision, Project administration, Methodology, Funding acquisition, Data curation, Conceptualization. **Margarida Maria Barros:** Writing – review & editing, Validation, Supervision, Funding acquisition, Conceptualization.

## Declaration of competing interest

The authors declare that they have no known competing financial interests or personal relationships that could have appeared to influence the work reported in this paper.

## Data Availability

Data will be made available on request.
